# The Tunisian experience of participatory health governance: the Societal Dialogue for Health (a qualitative study)

**DOI:** 10.1186/s12961-023-00996-6

**Published:** 2023-08-28

**Authors:** Hela Ben Mesmia, Dheepa Rajan, Rim Bouhafa Chtioui, Kira Koch, Imen Jaouadi, Hala Aboutaleb, Sana De Courcelles, Louisa Atmani, Blanche Pujos, Ali Mtiraoui

**Affiliations:** 1 Faculty of Medicine of Sousse, Research Laboratory “Quality of Care and Management of Health LR12ES03”, Doctoral Commission “Health Sciences”, University of Sousse, Sousse, Tunisia; 2 World Health Organization European Centre for Health Policy/European Observatory for Health Systems and Policies, Brussels, Belgium; 3 Independent UN Expert, Tunis, Tunisia; 4 World Health Organization Headquarters, Geneva, Switzerland; 5 High School of Business, Manouba University (ESCT), Manouba, Tunisia; 6 World Health Organization Regional Office for the Eastern Mediterranean, Cairo, Egypt; 7 Paris Institute of Political Science (Sciences Po), Paris, France

**Keywords:** Heath system reform, Health policy, Citizens’ participation, Health democracy, Universal Health Coverage, Societal dialogue, Health governance, Participatory governance

## Abstract

**Background:**

Tunisia has been engaged in the Societal Dialogue (SD) for Health process since 2012, a participatory health governance process aimed at bringing in people’s voice into health policy-making. Its first success was the recently released National Health Policy 2030. This paper aims to document the SD process and to bring out the lessons learned to inspire other countries.

**Methods:**

This study was based essentially on a qualitative analysis of semi-structured interviews with citizen jury members and health experts that took place from May to September 2018. The qualitative analysis adopted an inductive-deductive approach according to a cross-matrix between the themes of the interview of the two groups of interviewees.

**Results:**

The qualitative analysis of the data highlighted that the Societal Dialogue created a health democracy dynamic with inclusive dialogue spaces for the population, communities, and civil society to participate in health system design. It constituted a multi-actor and multidisciplinary coordination platform to increase consensus building among actors. Initial government support and high levels of volunteer commitment allowed the process to achieve a certain level of sustainability. However, this process faced and still faces many challenges such as overreliance on volunteers; a crisis of trust; political instability and the lack of an effective communication strategy. These challenges negatively influence the policy uptake of recommendations made by the Societal Dialogue for Health.

**Conclusion:**

The Tunisian societal dialogue experience highlights both the successes and challenges of a structured participatory platform, as well as the effort and perseverance it takes to keep such a process functional and relevant. A key lesson from this study is that this model of participatory health governance eventually reaches a stage where population, community, and civil society participation needs to be more institutionalized within the government routine so that it can credibly feed into health policy review processes and inform decision-makers on a regular basis.

## Background

In post-revolution Tunisia, addressing social inequalities and fostering more democracy became key population demands, ones which became more insistent as the economy deteriorated in the decade since 2011 [1]. In health, this meant more population, community, and civil society say into how the health system is shaped and how health system reform should play out. Amplifying people’s voices as an integral part of health system governance can help to elaborate more responsive policies that tackle key population issues in terms of access, quality, and user experience, paving the way towards building a people-centered health system [2].

The mismatch between the apparent performance of a fragmented health system and the growing expectations of society ultimately created enough internal pressures for health authorities and politicians to act upon new and persistent challenges (Cf. Box 1). Calls for needed and urgent reforms were made, in particular to address the significant disparity in service provision to the detriment of interior regions who face many infrastructure and staff resources distribution challenges [3].

Box 1: Tunisian health system main issues [3]

- a lack of access to health facilities and a lack of coordination between them,
- a failing quality and safety monitoring,
- a deteriorated public health sector and a private sector that is developing in a poorly regulated way,
- a high level of out-of-pocket contribution to the financing of health services,
- demotivated human resources.

In this context, and as part of responding to the increased public demand, he “Societal Dialogue for National Health Policies, Strategies, and Plans” (SD) was launched in Tunisia in 2012 (Cf. Box 2). Its stated aim is to initiate a transparent public debate with a participatory and inclusive approach (Cf. Box 3) and strengthen the role of the population and civil society in ensuring the right to health. The first major task of the SD was to involve lay people and civil society in the health system reform processes by organizing their structured input into the development of the first Tunisian national health policy (NHP).

Box 2: SD governance structure

- The SD governance structure is a mix between government and volunteers.
- The Ministry of Health (MOH) operates a dedicated administrative management unit for the SD but it is run as a MoH project rather than it being a government institution per se.
- The actual work of the SD is not primarily undertaken by government officials. Several working groups are coordinated by a Technical Committee which is composed of Ministry cadres, volunteer experts from civil society, professional associations, academia, and WHO. The steering committee has a similar composition and is chaired by the Minister of Health, its mandate being to monitor and validate of all the steps, methodology and outputs of the process.

Box 3: Participatory governance in health Participatory governance in health aims to increase citizen participation in public policy processes in order to meet the health needs of citizens and to improve access and quality of health services [4].

The SD process was designed in three phases (Cf. Appendix 1):

1. The first phase “The health system diagnosis phase” (2012–2014), consisted in building a common understanding of the challenges for the health system [5]. More than 3400 participants were involved in various participatory dialogue spaces discussing health system issues and proposing solutions that were put forward in the White Book (2014) [5].
2. The second phase “The NHP 2030 [6] development phase” (2016–2021) focused on defining the main strategic directions and content of the NHP. The NHP document was the result of collective and participatory work involving a total of 3263 participants, including experts, citizens, and health professionals to discuss and enrich strategic choices [6].
3. The third phase “The NHP implementation phase” (2021–onwards) is based on a participatory approach for monitoring and evaluation.

Tunisia has accomplished the first and the second phase of the SD process and a new process for NHP operationalization is currently in progress within the Ministry of Health. The government and civil society officially approved the NHP during the celebration of World Health Day, the 7th April 2021.

This unique process in Tunisia is yet to be documented scientifically and in a comprehensive way. This paper aims to discuss the SD process and highlight the main success factors as well as the challenges and bottlenecks, to understand through the perception of societal dialogue stakeholders what worked well and less well and to feed into and improve subsequent phases of the societal dialogue process.

This study is a crucial step towards drawing lessons and recommendations that will serve to enrich similar experiences in other countries on how to engage the population to contribute to health decision-making processes. It will also help Tunisia to further develop this participatory approach in order to best meet the needs and expectations of its population.

## Methods

### Study design

The approach used for this study is inspired by Sharan Merriam’s [7] view of a process description and analysis; which relies on qualitative data to understand the inner workings of a process, with a document review as an essential phase contributing to the research design.

Given that the Tunisian process was a long-term one which involved several phases culminating in health policies development, a longitudinal approach [8] was adopted in terms of examining the process over time. This method is most suitable for transitional periods’ [9]. The unit of analysis is linked to "events" by explaining the dynamics of the process [10] to identify the causes of the change [11].

Semi-structured interviews took place from May to September 2018. A document review was undertaken of all relevant national documents and reports produced by the SD process. In total, over fifty documents were reviewed which contained mostly government documents, meeting/event reports, minutes, technical reports developed by the technical committee of the SD and/or external consultants as well as WHO documents on the progress of the SD process and other technical analysis. These documents were collected from the archives of the Ministry of Health, the WHO Tunisia office, and the SD website www.hiwarsaha.tn.

### Participants and sampling

The semi-structured interviews were carried out for two groups of key actors involved at different levels of the process (citizen jury (CJ) members and experts). Purposive sampling was adopted according to preselected criteria: the active involvement in the SD (phase one and two) through participation in dialogue spaces for examples; the sound understanding of/familiarization with key deliverables produced by the SD process and reflecting the diversity of profiles as mentioned in the Tables 1 and 2. Sample size was not fixed prior to data collection and was adjusted according to the saturation of the data [12]. People who best met the criteria were first contacted. All the contacted people agreed to take part in the study.

**Table 1:** Experts group

Profil	Characteristics	Total
Academia	1 Public health specialist 1 Health economist	2
Health services provider	1 Public health facility practitioner	1
Civil society	1 Public health and right to health specialist 1 Public sector practitioner	2
Health insurance	1 Health economist 1 Public health officer	2
Health professional body	1 Private sector practitioner	1
Total		8

**Table 2:** Citizens’ jury group

Profil	Tunisia region	Total
Health professional	1 South-west	3
	1 North-west	
	1 South-east	
Civil society	1 Center-east (Sahel)	2
	1 North-capital (Tunis)	
Academia	1 North-east	1
Retired—lay citizen	1 North-capital (Tunis)	1
Total		7

Out of the 15 key informants interviewed, eight were experts belonging to the technical committee or the working groups of the SD and seven were citizen juries (four lay citizens and three health professionals). The key informants’ affiliations were government department, academia sector, healthcare facilities or civil society. The citizens juries involved were selected from the three regions of Tunisia (north, center and south).

### Data collection tool and procedures

An interview guide was used to elicit the views and experiences of interviewees on the SD process overall (phase one and two) regarding its results and methodology in particular public participation methods used, communication and future perspectives (Cf. Appendix 2). The language used for the interviews was French. The participants responded in French and in the local language which is a mix of French and Arabic. The transcriptions were then translated to French by an independent bilingual expert and the translation verification was done by the Tunisian researchers. The interview guide was piloted with 2 sample interviewees before finalization.

Each interview lasted between 30 and 60 min. All the interviews were audio-recorded after obtaining verbal consent and were conducted, according to the convenience of the interviewee, either at the Ministry of Health (office of the administrative management unit for SD) or in the WHO Tunisia office or by phone. Interviewees were assured of confidential analysis of their interviews. Each interviewee received an information note which contained the objective of the study and emphasizes the voluntary nature of taking part.

### Data processing and analysis

Data were transcribed verbatim. An inductive deductive coding and thematic analysis was undertaken according to a cross-matrix between the themes of the interview and the two groups of interviewees. The documentation review served at the beginning to build a theoretical coding framework. Then and as recommended by Merriam’s methodology [13], data collection and analysis were performed simultaneously. Several discussion meetings were conducted between the members of the research team to identify new themes for coding based on a content analysis approach.

The data collection was subject to a thematic analysis with reference to the work of Miles and Hubermas [14] following an open codification to highlight the different links between the concepts.

The analysis grid was broken down into context, actor, environment, and practices. The data analysis process followed different stages, starting with the prioritization of the interviews as guided by the work of Beaud and Weber [15]. This first consists in classifying the data according to the interest of each interview to identify those who will occupy a central place for the analysis by reference to the objectives and research problematic and which deserves to be deciphered and transcribed in an integral way.

For the sake of validity and reliability of the data on the one hand, and to reconstruct "a chronology of actions due to problems related to the memory of the respondents" [16], the research team analyzed the transcriptions using a data triangulation logic. The multidisciplinary team consisted of national researchers, WHO staff and independent external parties, thereby bringing together national expertise on the SD process as well as international expertise in health policy and participatory governance. Each transcript was coded by at least two researchers separately. Codes were discussed among the group to reach consensus [17], thereby reducing bias in subjectivity. For quality appraisal purposes, the methodology used for this manuscript was verified based on Consolidated criteria for reporting qualitative studies (COREQ) checklist [18] (Cf. Appendix 3).

## Results

*The SD has created a new dynamic to amplify people's voice in policy-making by providing a platform for dialogue and debate where communities and civil society were explicitly given a say.*

In the launching context of the SD, the major challenge was to find a way to change the old-style closed-door governance to a more modern, responsive approach, which ensured that health policies included people’s voice and responded to the needs of the population.

The huge gap between the needs and expectations of the population on the one hand and the vision of the government on the other hand underscored the raison d’être of the SD. With the creation of a platform where people can come together and deliberate upon pressing health systems issues, this idea was made concrete amidst great hope.

This new multi-actor dynamic for health system reform as described enthusiastically by one of the interviewees reflected what a majority of interviewees also expressed:

"I think it is a very rich, very enriching, very innovative process that meets, let's say, the expectations of the population … it is in favor of all stakeholders, so it meets a need” (expert, Medical Association representative).

The bottom-up dynamic in contrast to the top-down decision-making style which had existed for decades is reflected on by these interviewees: “[Before], everyone was expected to think what the people up there think… we [didn't] have the right to criticize or give our ideas. And that is what has changed in this SD, that is to say that the citizen has been introduced into this way of managing, let's say, this health reform” (health professional).

“I think that there are […] people who will carry the voice of the citizens, who will discuss and defend their point of view and negotiate it” (Expert).

The SD process placed a particular emphasis on the ‘societal’ component, highlighting that the entirety of society’s actors should have a say in how their health system is shaped. To this end, various participatory spaces were created (see Table 3) to allow for lay people, community groups and civil society to express themselves. The open-minded spirit of these spaces was conveyed by one of the interviewee’s comments: “let the citizen say what he/she wants to say” (CJ member, health professional).

**Table 3:** Dialogue spaces during the SD process

Participatory space	Description
Regional Meetings on Health	These meetings were usually organized at the regional level, by invitation and sought ‘societal’ input on specific health topics. Experts put preparatory material together beforehand. Especially civil society opinions and views were sought on precise, more technical questions
Open mic sessions	These meetings aimed at hearing from all parts of society and touched up more general, overarching health topics such as what the future health system should ideally look like
Focus groups	They were set up with communities that were not participating in other participatory spaces. Marginalized and vulnerable groups were thus targeted in this small-group, homogenously constituted sessions
Citizen’s jury	The CJ were drawn on the day of the meeting according to the basis of voluntary presentation to the draw to be part of the CJ representing the region with the task of pronouncing a verdict on specific questions linked to specific themes
Thematic working groups with targeted consultations with communities and civil society	While thematic groups largely constituted of thematic experts, a concerted effort was made to engage with affected communities and civil society actors to feed into evidence analyses
National Health Conference	A large participant list including CJ members, associations, NGOs, trade unions, parliamentarians, and many others come together here to validate policies and decisions

All types of interviewees, for example academia, civil society stakeholders, and lay citizens, praised opportunities to discuss the issues and dysfunctions at both the regional and local level, allowing for spaces beyond the capital where decisions were traditionally taken in the past: “The SD… it’s when we find ourselves around the table together to jointly identify a matter of concern and [then] to exchange on the different points of view with the hope of reaching a consensus to conclude on the matter of concern” (Expert, public health, academia).

Also lauded was the broad outreach to the wider population at large as described in this citation, which included population groups whose voices are usually less heard: “We follow the training, we sit in cafes, in bars, in the metro, on the bus, anywhere, talking to old people and talking with women"(CJ, lay citizen).

*This new SD dynamic also provided a novel and participatory approach to health planning and health system reform*

The SD process brought to light the multiple challenges the country faces such as health system deterioration, a bureaucratic culture, and health inequity due to disparities and poor distribution of human resources and medical products. These challenges were acknowledged by all interviewed stakeholders as urgent, necessitating the active involvement of the population in addressing them within the health planning & policy process:

"Firstly, we find ourselves in a situation where we urgently need to develop and promote the [health] sector, in service of citizens and their health, and to serve the general public interest" (CJ, health professional).

“Given each person’s unique perspective, each one of us will express what he expects from this reform, as well as how he/she sees the reform… Especially practitioners on the ground should participate in this reform” (health professional association).

The active involvement of the population in health planning meant a new way of working—interviewees felt that this was precisely what the SD approach offered:

“[The SD] is an innovative process that has an enrichment for planning … the interest of such a process is that it develops a vision, a strategy, and programs to implement this strategy, and achieve this vision” (Expert, health economist).

“I think that the participatory process is the solution, given the socio-political context in the country, it is the solution which helps reach effective decisions—then even when they are painful, they will be accepted, because they are enlightened enough, informed enough and well adapted to the context of the decision… it is a vital process for the reform in Tunisia.” (Expert, public health).

The first-ever participatory situation analysis released through the White Book in 2014 [3], and the adoption in 2021 of the first NHP 2030 [4] outlining a joint vision for the sector are both widely seen as SD products; with them, the SD’s influence on key policy documents is evident.

“With experts and SD committees, with the input of associations and representatives of civil society, we addressed the problems and failures by presenting solutions and proposals that are formulated in the white book” (CJ, health professional).

*The new participatory dynamic was a clear achievement in and of itself in many ways, given the stark background of a crisis of trust between government and its population.*

The post-revolution period was marked by deep mistrust among stakeholders in general, and Tunisia was not spared. Throughout the SD process, a key challenge was stakeholders’ skepticism as to whether a real opportunity existed to collaboratively identify challenges and policy solutions to improve population health.

This crisis of trust around the process was perceived at three main levels:

(i) The context in which the SD process was initiated was marked by mistrust between civil society and politicians:

“There are a lot of tensions both between and amongst politicians [and] civil society… the image [of in-fighting] that parliamentarians gave to the time was not a favorable factor [for dialogue]» (Expert, public health, academia).

(ii) A crisis of trust between people and health professionals and by extension, the health system, mainly due to the lack of health service quality and accessibility:

“It was expected, tensions between professionals and citizens…lack of trust in the system, we complain about the doctor who comes late…the appointment lead times are long…[leading to] legitimate tensions” (Expert, health economist).

(iii) A considerable public distrust toward the ministry of health:

“Two days ago, I heard on the news [that] the Minister of Health [was] say[ing] that he [can] help…Guinea in the field of health… I would like to tell him that he should first work here, that he should first work in Tunisia and only afterwards [look beyond], on an international scale” (CJ, lay citizen).

The participatory approach of the SD was expressed to be an important element to tackle these trust issues by particularly giving people and civil society more agency and awareness over health issues:

« From our side, when we arrived at the SD, we returned with confidence» (CJ, health professional)

Several interviewees from civil society as well as health professionals shared the impression that trust in the system had improved through the SD process:

« It was this participatory democracy that made it possible … even with all the disagreements that took place, people were hopeful that things were moving forward and that (…) the process was in the right gait, let's say, on the right line." (Health professional body representative).

“Over time, these same people, these associations, this civil society who continued to participate to finalize SD deliverables, they are somewhat confident that they participated in setting up a system that can meet their needs.” (Health insurance fund representative).

*The SD platform benefitted from a multitude of actors which fostered multi-disciplinary thinking and engagement with a broader group of actors than is normally the case.*

The SD process laid a great emphasis on inclusiveness with targeted outreach and involvement of a large number of stakeholders: lay citizens, civil society representatives, unions, health professionals, academia, and decision-makers including the Prime Minister’s Office, parliamentarians, Ministry of Health and other related ministries. Bringing in a broad range of actors allows for drawing from the various expertise areas to examine a problem from different angles, thereby increasing the quality of discussions and decisions.

“The multidisciplinary aspect was very present during the process, not only doctors are involved but we see that other actors participate: lawyers, managers, economists, pharmacists, and civil society, lay citizens too; such involvement is the basis of the wealth created around the discussions of the different themes”. (Expert, health economist).

In addition, multi-disciplinarity created space for, and explicitly valued, experiential knowledge and expertise in addition to expert- or research-based knowledge. This is demonstrated in the specific outreach, especially at the beginning of the SD process, to ensure that as many population groups, however remote and reticent they were, had a chance to express viewpoints.

For example, homogenous focus groups were a response to the observation that women and vulnerable population groups were not attending nor speaking up at larger forums and meetings. Focus groups with patients living in remote areas, with single mothers, with isolated senior citizens, and with families living in polluted industrial areas, were organized in the interior of the country, in community settings, away from the coast and Tunisia’s capital. Both the setting and comfort of being ‘among peers’ allowed for more frank discussion as well as a deeper understanding of the varied challenges these groups faced in interfacing with the health system.

*A core group of committed volunteers and good initial government support on the principle of participation contributed a great deal to the SD’s success. Yet the limits of relying on volunteers mitigated that success, as did the political upheaval of the subsequent years following the SD’s launch.*

The SD’s large-scale effort to foster population participation was linked to the considerable amounts of volunteer time put into the Steering Committee, Technical Committees, and Working Groups, showcasing the commitment and motivation of multiple actors to join a reform project to improve their country’s health system. One expert summed it up as such:

“One of success factors was that we built the process on volunteering taking into account different profiles: health professionals, experts and civil the society representatives “ (Expert, public health).

The commitment of a core group of stakeholders in SD governance and management reflected the deep desire at the time to change the status quo:

“There are people who want to work, and… push for SD” (CJ, health professional)

“People worked night and day” (CJ, health professional)

However, relying on the professional and social commitments of SD volunteers was challenging in terms of sustaining it in the long-term:

"There are a lot of people who have started with us, who lost their motivation in the meantime and therefore we are not enough to continue” (Expert, Public Health facility practitioner).

Compounding this were high population expectations and the challenges of navigating the complexities and realities of government policy-making to ensure SD results were adequately considered and taken up:

“Due to these periods of stagnation, the participation of the various stakeholders is insufficient” (Expert, civil society)

“People are getting tired …. because there is no political will” (CJ, Civil society)

Nonetheless, a group of hard core volunteers, mainly those in the Technical Committee, persisted and continued to ensure the continuity of the SD, and to nourish, as far as possible, a climate of trust despite the constraints related mainly to political instability:

“[We] kept the technical committee at an equal distance to all the actors, [i.e.]the ministries, the unions, the associations and the experts, it was not easy, it was not easy at first, but it was built up over time, I think it was a good thing.” (Expert, public health).

“The technical committee is practically apolitical, … so we are considered at equal distance with everyone and we have tried to preserve this advantage” (Expert, health economics).

*A lack of clear and consistent communication regarding the SD was also a key constraint, mitigating the SD's achievements*

Communication on the nature of the SD process, its objectives and aims, and whom it plans to reach and include, was critical within the post-revolution context of mistrust in a country with a previous history of tokenistic consultations. The SD lacked the capacity, with its volunteer base, and without huge funding, to have a well-reflected communication strategy put together by communication professionals.

Nevertheless, within its limited means, the SD partnered with the media to allay skepticism of a tokenistic approach, sharing detailed information on SD meetings and its participatory events. In an attempt to ensure accurate messaging to the public, the SD organized training workshops for media representatives. These workshops equally served as a platform to answer and clarify questions around the SD process.

The media showed much interest in the SD process, with television and radio stations reporting on the SD and interviewing SD stakeholders. This helped raise awareness and conveyed some of the SD results to a broader audience. However, the lack of a communication strategy to support media interventions meant that its impact was limited:

"I would have liked national television to cover everything … it's a very important thing...to encourage the citizen [to participate] (lay citizen member of the CJ)".

One CJ member lamented that even though communication is considered part of their role, “the CJ does not have the means it needs to transmit information to [other] citizens (CJ)”. The CJ members nevertheless did what they could in recounting their experience, explaining the SD process, and publicizing its events within their personal, and other, networks. This was especially crucial during the long gap in events after 2014 due to political tumult.

*The above-mentioned challenges greatly limited policy ownership and uptake, despite the many successes of the SD initiative which should be built upon and learnt from.*

At the beginning, the participatory approach of the SD process led to great hopes that health decisions would then enjoy all-round buy-in by stakeholders:

“The [SD] process facilitates buy-in of a [policy] decision» (Expert, public health)

However, as time passed, and the SD process took much more time than initially expected, it became clear that long periods of political upheaval negatively influenced political will of the various health ministers in place. The instability of ministerial positions (eleven ministers in total during the period 2012–2021) exacerbated the myriad other challenges mentioned earlier, leading to periods of low ministerial ownership of the SD process:

"Every time there is a new minister, you start again from the beginning, you talk to him about the SD and all that it contains, perhaps we must even insist on convincing him (CJ, health professional) "

“The risks are related to political ownership [of the SD], at the decision-making level, due to the change of ministers” (Expert, health economist).

One long gap period for the SD came after launching the participatory situation analysis report of the health sector in 2014; the frequent changes of ministerial department heads lead to a subsequent slow pace of implementing SD recommendations. In the eyes of the many stakeholders, including the public, this weakened the SD’s nascent credibility:

“The reasons are linked to political instability,…the gap took place because at that time the government and our country was not stable, there were many changes of governments” (CJ, lay citizen).

"As a process … I see it is very important…unfortunately it has stagnated” (CJ, lay citizen)

“The fact that it has been dragging on so slowly since 2012, it somewhat diminishes [the SD’s] credibility” (Expert, civil society)

One civil society participant underlined that “[p]eople are getting tired… because there is no political will”. He regretted further that the SD for Health proposals “could not have [the attention of] the ministry. I was a member in several workshops and in reality, we got tired”.

One of the many reasons for low policy uptake of SD results might have been a lack of negotiation and the late involvement of some actors, mainly unions and political parties:

“The focus of participation was linked mainly to citizens, associations, local actors….trade unions and political parties were [only engaged with] at the end of the first phase … in the national health conference “ (Expert, health professional body).

“[The SD process only] partially [meets my expectations], first, because of these periods of stagnation, … and on the other hand, …participation of the various stakeholders is insufficient” (Expert, civil society).

Another possible reason for the low policy uptake was that some mid-level government cadres saw the SD process rather as a threat and not as complementary to their policy work. Much of this was based on resentment due to the unclarified link between the SD’s planning input and the Ministry’s health planning work at the time.

“There was resistance from the administration [of the Ministry of Health, because] ….the health planning process was done from an external group” (Expert, public health).

## Discussion

In nearly a decade since the SD’s existence, the Tunisian experience has brought to light the multitude of challenges and learnings on its path towards a more participatory and inclusive approach to health decision making. It thus adds to building an evidence base on a large-scale participatory governance effort at national level, initiatives which have been rarely studied, especially in low-and middle-income countries. This work thus facilitates cross-country learnings by outlining the pathways and lessons learned by Tunisia after a relatively long experience. As laid out through the various phases of the SD process, that pathway is not a straightforward one. Rather it is a nonlinear process with ups and downs which requires the need to constantly adjust and adapt. The revolution context provided the window of opportunity to stimulate a new way of working but embedding such a participatory approach into health decision making practice has been a constant issue of concern. Institutionalizing the SD process as a legitimate approach to amplify people’s voice in health decision making is one way forward. While there may be caveats, it should be reflected on thoroughly, as discussed in the following paragraphs, in terms of how to sustain participation as a modus operandi into the future.

### Ensuring participation in health decision-making is integral to the right to health

The concept of the right to health is inextricably linked to participation. As first put forward by WHO’s Constitution in 1946 [19] and followed suit by numerous national constitutions and various treaties, the right to health manifests the notion that for people to realize their right to health, they must have a say in it. How this right is realized in practice and claimed by people varies considerably among countries though.

In Tunisia, social participation is seen by many citizens and civil society activists as a constitutional right, recognized within society, and claimed through the SD process. The SD is thus perceived to be a major step in the direction of more health democracy to better respond to population needs when it comes to health care. Through the participatory spaces facilitated by the SD process, citizens have the possibility to claim participation rights and express viewpoints and demands. In turn, this means equally for governments to establish, foster, and maintain those participatory spaces for this dialogue to happen—and this, importantly, not only sporadically but regularly over time.

However, as the results demonstrated, the SD process has been especially volatile to political upheaval and changing government commitment to invest in the process. Yet again, the SD process has been kept afloat since a decade, benefitting from active, structured participatory activity undertaken by civil society actors with fluctuating support from decision-makers. The SD process can thus be seen as a fertile ground and it’s about time to use this opportune momentum to bring in legal protection to this process to enshrine the right to health in Tunisia’s health decision-making practice.

### Embedding the SD into a legal framework can help make it more resistant against political turmoil

Embedding the SD process into a legal framework is one option to foster continuity over time. Increasingly, this is a demand put forward by key Tunisian stakeholders as a counterbalancing act to be more resistant against political turmoil. Having a legal framework in place can be advantageous in many ways.

A legal text can protect the existence of the SD process for people and communities to claim (theoretically) their right to dialogue with governments on policy-relevant health topics. Second, a legal framework embeds the SD process into the health sector modus operandi, thereby facilitating the necessary ownership and institutional links to health and decision-making. The legal text would simply reinforce what is already acknowledged by a broad range of actors as a legitimate tool for strategic planning and health decision making. Third, undergoing such a process of elaborating and negotiating a legal framework is often inherent with a boost to the SD process and the political commitments behind it.

Nonetheless, it is important to keep in mind that a legal framework does not guarantee participation per se as it is one of several elements needed to build a culture of participation. For example, Portugal’s National Health Council only came into full operations in 2017, almost 25 years after the Basic Health Law serving as its legal framework was passed, because the political and other conditions for setting up the new institution were not in place [20]. Moreover, the devil is often in the detail. Legal texts need to be elaborated cautiously in terms of clarifying roles, mandates, budgetary implications, so that it does leverage and legitimize population voice, rather than reinforcing existing hierarchies and power dynamics with the countervailing consequence of the local elite capturing legally mandates spaces [21] instead. An example from Ghana’s Community-Based Health Planning and Service programme drives home this point, where pre-existing community structures reinforced male-dominated community leadership. Coupled with a vertical management style, the programme ended up leaving many woman and young people behind [22].

Experiences from Thailand [23] however demonstrate that legal frameworks such as the National Health Act of 2007 were vital in commencing a more institutionalized and inclusive culture of participation, anchoring into law and better connecting the many local participatory efforts that already existed before. It not only provided a boost in visibility but also in terms of resources, resulting in the establishment of the National Health Commission Office (NHCO) [24] which is given the legal mandate to run the yearly National Health Assembly. This could equally be the case for Tunisia’s SD steering committee and the Ministerial Unit in place to support the SD process [25], guaranteeing sufficient human and financial resources to fulfill a given mandate.

*Building a culture of participation and trust while mitigating power imbalances to ensure sustained engagement over time*

While legal frameworks can help to sustain participatory processes, there are many other factors that need to be put in place for the successful implementation of such frameworks to cultivate a culture of participation and trust. Given the decade of SD existence already, many of these factors are already in place in Tunisia; it is thus a truly opportune moment to collectively examine the legal framework option.

Yet, room for improvement exists. Above all, as repeatedly highlighted by many interviewees of this study, this means addressing power imbalances that are deeply entrenched in Tunisia’s social, political, and cultural landscapes, resulting in large mistrusts among actors to jointly work towards ‘better health for all Tunisians’ as laid out in the White Paper’s vision.

These power imbalances that influence all kinds of participatory spaces from regional townhall meetings, over citizen juries to national health conferences, are difficult to overcome. This is a constant challenge seen across all countries. Despite France’s long trajectory in health democracy, dialogue spaces still mainly attended captured by well-capacitated and resourced NGOs and lobby groups with very few lay citizens and members of vulnerable groups [26, 27]. Acknowledging power imbalances in the first place and subsequently mitigating them, first and foremost by being transparent and explicit about conflict of interests, is key to enabling, as far as possible, a level playing field for all actors to equally participate and influence policy. The SD has been a crucial process to amplify people’s voices, but also highlighted the inherent power struggles between health professionals, citizens and policy-makers.

Attention to format and design of SD-facilitated participatory spaces will remain key in this regard. For example, allowing for new voices to enter the SD steering and technical committees would address concerns that a small group of active stakeholders are dominating the SD, impeding others to join and influence debates. A rotating system is for example used by Portugal’s National Health Council for the six out of 30 seats reserved for civil society organizations; this provides the opportunity every four years for new groups to sit on the Council [26]. Here, analyzing who is participating in organized participatory dialogue spaces and who is not participating and subsequently reaching out to the latter, often the most marginalized groups, is pivotal. If, in this study, Tunisians no longer feel represented, the SD process risks losing credibility and influence. In this regard, a targeted communication strategy is much needed to raise awareness of what the SD process can do and not do, as many citizens’ felt keenly disappointed, partly due to goals and expectations which were likely not realistic in the first place. Being explicit about roles and mandates also helps to set and achieve realistic targets, which, in turn, keeps citizens’ motivation up to stay engaged.

Visible efforts from policy makers to consider population demands and feedback on how participatory results were taken up in policies and strategies will be equally important. Thailand for example established a follow-up committee to ensure resolutions that were passed in the National Health Assembly are being adequately taken forward and implemented as policy uptake has been a constant issue of concern [23]. The NHCO is taking up this coordinating role; but for them to operate successfully, it required heavy investments in government capacities. In particular (i) to recognize that participation is an added value to their policy work, (ii) to foster technical skills how to manage participatory spaces meaningfully while dealing with conflict of interests as well as (iii) to improve communication skills with the population, communities, and civil society [28]. All of this can help to foster a real culture of participation based on trust, transparency, accountability and a joint vision where people’s voice is firmly heard and legally embedded into health decision making. Especially trust is something that one needs to invest in constantly and which can be easily lost—which almost happened during Tunisia’s periods of political upheaval. Hence ensuring the long-term sustainability of the SD should be a political priority.

### Limitations of the study

This study is the first of its kind that attempts to document and analyze the SD process in Tunisia however several limitations exist. First, the data of this research is mainly based on participants experience and perceptions and are collected from a small sample which does not allow for generalization of findings. However, it does draw policy-relevant and interesting insights into the SD process due to key informant extensive experience and knowledge of the SD process. Second, interviews were conducted in 2018, noting that the SD process has been ongoing from 2012 until now. Due to the Tunisian political situation, with particular sensitivities around the SD process at the time, and then delays with the Covid crisis, it was not possible to publish the results earlier. Nevertheless, the same challenges highlighted in this article persist, and are thus highly relevant for the current context. Third a recall bias of participants needs to be acknowledged at the time of interviewing. Fourth, interviews were conducted among two groups: experts and citizen jury members; however not with politicians and policymakers. Another study examining the viewpoints of said group would be an interesting complement to this research study. In addition, the study can provide future avenues for more in-depth research that delves into specific issues touched upon in this article such as representation, capacities and policy uptake.

## Conclusions

Overall, the SD was highly appreciated by the participants of the study. However, it drew a lot of criticism in terms of its slow pace, the instability of political will, the difficulty in policy-maker ownership and integration into routine planning processes, the lack of a visible impact, and sustaining the motivation of the population as well as involved stakeholders over time. These critiques are still precisely the ones which are repeatedly raised now, for which the SD Steering Committee is grappling to find answers to.

Nevertheless, the SD process remains an innovative experience for the development of health policies based on a participatory and inclusive approach. Phase one of the SD enabled the production of the first-ever participatory analysis of Tunisia’s health sector, including a situation analysis and strategic orientations. The SD’s second phase produced the first national health policy 2030.

This study was useful to identify the key success factors and challenges of the SD process, according to the perception of participants, and drew valuable learnings not only for Tunisia but also for other countries on how to more meaningfully engage with the population at national level in shaping health policies and reforms. Much of the current literature on population and community engagement draws on sub-national, local, or specific programmatic experiences. Here, we have appraised a national-level dialogue which has sustained the test of time to stay in active existence for 11 years now.

A key success factor from Tunisia’s SD experience was the motivation of people involved that sustained the process for more than a decade. The inclusiveness of the process, citizen participation, good communication are also crucial factors to carry out this process. Finally, without political will and the effective involvement of Ministry of health cadres, the results of this process will face challenges in policy uptake.

## Data Availability

All data used and/or analyzed during this study are available from the corresponding author on reasonable request.

## Appendix 1: Summary of the description of the process of the Societal Dialogue for National Health Policies, Strategies, and Plans

### SD Governance structure

The governance structure established for the SD process is defined by legal texts [25] and organizational procedures. The SD structures are mandated to:

- Organize a consultative and consensual process including seminars, conferences, as well as other forms of events and dialogue spaces (see Table 3 of the manuscript for more information) on the various strategic and operational aspects to develop the health policy,
- Collect, update, and analyze all available data regarding the population health and the national health system,
- Develop strategic and operational options for the new health policy,
- Contribute to the sectoral development plans related to health in view of advancing sustainable development goals.
- These structures consist of a steering committee, a technical committee (TC), working groups and an administrative management unit.
- The steering committee

The steering committee is chaired by the Minister of Health and brings together executives and representatives of the Ministry of Health and other Ministries, political elected officials (Assembly of People's Representatives), professional organizations and unions, medical councils, training/academic institutions and scientific associations, civil society representatives, elected citizen juries and international partners (WHO and European Union).

Equal representation along the three main stakeholder groups (1/3 government cadres, 1/3 civil society and 1/3 academia including unions and professional associations) was ensured to guarantee the principles of dialogue.

The steering committee is responsible for ensuring the smooth running of the national dialogue spaces and approves based on the principles of consensus seeking the strategic orientations of the health policy put forward by the TC.

- The technical committee

The TC is made up of about thirty people with a core group of 5 to 10 dedicated personnel. It is composed of the president, the vice-president, the reporter, the responsible officer in charge of civil society coordination, the head of the administrative management unit and other experts who are chosen based on their skill sets, including representatives of ministries, professionals, and civil society.

The TC is responsible for carrying out the technical work of the SD process by ensuring the proper preparation, organization and running of the SD process at the national and regional levels, and for reporting technical outputs and state of progress to the steering committee. It also plays a coordinating and convening role between the authorities and the various stakeholders.

This committee is supported in its duties by the administrative management unit and working groups.

- The administrative management unit (AMU)

Administrative and logistical support for the organization of all SD events and meetings is provided by the AMU in collaboration with the WHO Tunisian office.

The AMU was created within the cabinet of the Minister of Health and is responsible for providing the secretariat and all the human and material resources required to enable the TC and working groups to accomplish all assigned missions in the best possible way within given timeframes.

The AMU consists of the president of the unit, a coordinating doctor, a coordinating administrative officer, a secretary, and a reception officer.

- Working groups

The SD working groups were set up according to themes that were agreed for each phase of the SD process. Based on those, initial technical reports were elaborated which were then adapted to suit population consultations. The number of working groups and themes were put forward in respective roadmaps prepared at the beginning of each phase by the TC in coordination with civil society representatives.

Working group compositions included all interested stakeholders including government representatives, civil society, health professionals and experts.

### The SD process organization framework

Citizen participation is at the core of the SD process. For this reason, the process “policy dialogue” was renamed to “Societal dialogue”, and by doing so, underscoring that the process encompasses not only the traditional way of conducting expert consultations and stakeholder conferences but also a regular and constructive dialogue directly with the citizens. This was done:

- For the analysis of the health system issues (SD phase 1),
- For the choice between alternative policy options (SD phase 2), and
- Later for evaluation (in the SD phase 3).

Eliciting perceptions and expectations of the population was thus a central piece of information to influence policy dialogue and debates, and thereby it was recognized as an integral part rather than a separate process.

The SD process was built around the engagement of three stakeholder groups: policy-makers, experts and professionals, lay citizens/patients, through adequate channels to involve each group, either through their representatives, or via direct engagement (lambda citizen and lambda professional).

Each phase of the SD process was structured in five major steps: expert phase, debate phase, deliberative phase, consensus phase and political adoption. These steps were not organized one after each other but a rather in a repeated and iterative manner as needed.

- *Expert phase* was based on the working groups who prepared technical guidance notes for each topic covered. This phase considers also the technical work done by the TC after each step for purposes of harmonizing, reporting and drafting documents. If needed, the TC was supported by international experts who provided advice and external reviews of the SD documents.
- *Debate phase* was based on population consultations. It aims to capture the perception of citizens and their expectations with regards to the technical work that emerged from working groups and presented by TC members (health system issues in the SD phase 1 and NHP strategic choices for phase 2 of the SD). The debate phase adopted several dialogue mechanisms and spaces (focus groups, open MIC in phase 1, regional meetings). These population consultations were done in each governorate of Tunisia. In the first phase of the SD, citizens were recruited directly from the general population; this was done with the support of medical students who gathered in public spaces and invited random people passing by to participate in the meetings, while looking out for a good mix according to age and gender. The selection criteria used to recruit citizens in the second phase of the SD’s population consultations were addressed in advance by the TC in order to ensure a balance based on population characteristics such as age, gender, locality. The recruitment and invitations to meetings were supported by the regional health department in each governorate in coordination with the citizen’s juries of the first phase who played a coordinating role with local communities. In each meeting over 100 participants were present. The regional meetings gathered not only lay citizens but also local associations and health professionals.
- *Deliberative phase* was done directly after the debate phase. From each regional meeting, four citizens were elected by lottery to be part of the citizen jury including 2 entitled and 2 substitute persons (24 governorates, 96 citizens’ jury in each SD phase). The elected Citizens’ Jury (CJ) members were then brought together to draw up their recommendations on the SD main documents (white book for the first phase, the NHP for the second phase), with inputs feeding into the National Health Conference.
- *Consensus phase* aims to obtain and build stakeholder engagement. It was run mainly during the National Health Conference (September 2014 in the SD phase 1, June 2019 in the SD phase 2). This conference gathered all the health system stakeholders (over 400 participants) who expressed their commitment in adopting the SD results and documents. During the conference, citizen jury members presented their recommendations. The consensus phase also encompassed advocacy work done during the SD process.
- *Political adoption* was obtained after the official adoption of the SD documents/deliverables by the government. Development of legal frameworks and operational plans based on SD documents is part of this step.

## Appendix 2: Interview guide

### I—Global overview of the SD process

1. How were you involved in the Social Dialogue (SD) process on health? Since when have you been involved?
2. How do you see the importance of such a process in Tunisia?
3. Do you think that the approach taken is adequate?
4. What were your expectations at the beginning of the process? Were expectations met?

### II—Phase 1

1. Regarding the process of phase 1 of the SD; what is your opinion about the methodology and mechanisms used in general?
2. What do you think about the methods and mechanisms used for citizen’s participation during this phase? Was there sufficient citizen representation?
3. Were you well informed about the progress and the results of this phase (through SD website, medias, social networks, etc.)?
4. What is your opinion on the main outputs of this phase (“White book” and “Declaration of the national health conference”)?

### III—The gap between phase 1 and 2

1. Phase 1 ended in September 2014 and phase 2 started during 2016. What do you think were the reasons for this gap?
2. Do you have an idea what was done between phase 1 and 2?

### IV—Phase 2

1. Regarding the process of phase 2 of the DS; what is your opinion about the methodology and mechanisms used in general?
2. What do you think about the methods and mechanisms used for citizen’s participation during this phase? Was there sufficient citizen representation?
3. Were you well informed about the progress and the results of this phase (through SD website, medias, social networks, etc.)?
4. What is your opinion on the strategic choices discussed for the project of the National Health Policy 2030?

### V—Perspectives

1. Do you know how the SD process will continue after the end of phase 2?
2. Do you consider it necessary for such a process to be continued in Tunisia?
3. How do you see the future of this process in Tunisia?

## Appendix 3: Consolidated criteria for reporting qualitative studies (COREQ): 32-item checklist

**Table:**

No. item	Guide questions/description	Reported on
Domain 1: Research team and reflexivity		
*Personal characteristics*		
1. Inter viewer/facilitator	Which author/s conducted the interview or focus group?	Page 23
2. Credentials	What were the researcher’s credentials? E.g. PhD, MD	Page 1
3. Occupation	What was their occupation at the time of the study?	Page 1
4. Gender	Was the researcher male or female?	Page 1
5. Experience and training	What experience or training did the researcher have?	Page 1
*Relationship with participants*		
6. Relationship established	Was a relationship established prior to study commencement?	Pages 4, 23
7. Participant knowledge of the interviewer	What did the participants know about the researcher? e.g. personal goals, reasons for doing the research	Page 4
8. Interviewer characteristics	What characteristics were reported about the inter viewer/facilitator? e.g. Bias, assumptions, reasons and interests in the research topic	Pages 4, 5, 6
Domain 2: study design		
*Theoretical framework*		
9. Methodological orientation and Theory	What methodological orientation was stated to underpin the study? e.g. grounded theory, discourse analysis, ethnography, phenomenology, content analysis	Page 4 and 6
*Participant selection*		
10. Sampling	How were participants selected? e.g. purposive, convenience, consecutive, snowball	Page 4
11. Method of approach	How were participants approached? e.g. face-to-face, telephone, mail, email	Page 6
12. Sample size	How many participants were in the study?	Page 4 and 5
13. Non-participation	How many people refused to participate or dropped out? Reasons?	Page 4
*Setting*		
14. Setting of data collection	Where was the data collected? e.g. home, clinic, workplace	Page 6
15. Presence of non-participants	Was anyone else present besides the participants and researchers?	Page 6, only the interviewer and the participant were present (one to one interviews)
16. Description of sample	What are the important characteristics of the sample? e.g. demographic data, date	Page 5
*Data collection*		
17. Interview guide	Were questions, prompts, guides provided by the authors? Was it pilot tested?	Appendix 2 and page 4
18. Repeat interviews	Were repeat interviews carried out? If yes, how many?	No, page 4
19. Audio/visual recording	Did the research use audio or visual recording to collect the data?	Audio recording, Page 6
20. Field notes	Were field notes made during and/or after the inter view or focus group?	No, Page 6
21. Duration	What was the duration of the inter views or focus group?	Page 6
22. Data saturation	Was data saturation discussed?	Page 4
23. Transcripts returned	Were transcripts returned to participants for comment and/or correction?	No, Page 6
Domain 3: analysis and findings		
*Data analysis*		
24. Number of data coders	How many data coders coded the data?	Pages 6, 23
25. Description of the coding tree	Did authors provide a description of the coding tree?	Page 6
26. Derivation of themes	Were themes identified in advance or derived from the data?	Page 6
27. Software	What software, if applicable, was used to manage the data?	NA
28. Participant checking	Did participants provide feedback on the findings?	No, Page 6
*Reporting*		
29. Quotations presented	Were participant quotations presented to illustrate the themes/findings? Was each quotation identified? e.g. participant number	Page 6 to 11
30. Data and findings consistent	Was there consistency between the data presented and the findings?	Yes, Page 6 to 12
31. Clarity of major themes	Were major themes clearly presented in the findings?	Page 6 to 12
32. Clarity of minor themes	Is there a description of diverse cases or discussion of minor themes?	Discussion of major and minor themes From page 11 to 14

## Abbreviations

CJ: Citizen Jury
NHP: National Health Policy
SD: Societal Dialogue
WHO: World Health Organization
NHCO: National Health Commission Office
TC: Technical Committee
AMU: Administrative Management Unit
